# CU06-1004 (endothelial dysfunction blocker) ameliorates astrocyte end-feet swelling by stabilizing endothelial cell junctions in cerebral ischemia/reperfusion injury

**DOI:** 10.1007/s00109-020-01920-z

**Published:** 2020-05-16

**Authors:** Dong Young Kim, Haiying Zhang, Songyi Park, Yeaji Kim, Cho-Rong Bae, Young-Myeong Kim, Young-Guen Kwon

**Affiliations:** 1 Department of Biochemistry, College of Life Science and Biotechnology, Yonsei University, 50 Yonsei-ro, Seodaemun-gu Seoul, 03722 Republic of Korea; 2 CURACLE Co., Ltd, Gyeonggi-do Seongnam-si, Republic of Korea; 3 Department of Molecular and Cellular Biochemistry, School of Medicine, Kangwon National University, Gangwon-do Chuncheon-si, Republic of Korea

**Keywords:** Ischemia/reperfusion injury, Blood‑brain barrier, Endothelial cell, Astrocyte end-feet swelling

## Abstract

**Abstract:**

Cerebral ischemia, or stroke, is widespread leading cause of death and disability. Surgical and pharmacological interventions that recover blood flow are the most effective treatment strategies for stroke patients. However, restoring the blood supply is accompanied by severe reperfusion injury, with edema and astrocyte end-feet disruption. Here, we report that the oral administration of CU06-1004 (previously Sac-1004), immediately after onset of ischemia/reperfusion (I/R), ameliorated cerebral damage. CU06-1004 stabilized blood‑brain barrier by inhibiting the disruption of the tight junction-related protein zona occludens-1 and the cortical actin ring in endothelial cells (ECs) after I/R. Interestingly, CU06-1004 significantly suppressed astrocyte end-feet swelling following I/R, by reducing aquaporin 4 and connexin 43 levels, which mediates swelling. Furthermore, the degradation of β1-integrin and β-dystroglycan, which anchors to the cortical actin ring in ECs, was inhibited by CU06-1004 administration after I/R. Consistently, CU06-1004 administration following I/R also suppressed the loss of laminin and collagen type IV, which bind to the cortical actin ring anchoring proteins. Unlike the protective effects of CU06-1004 in ECs, astrocyte viability and proliferation were not directly affected. Taken together, our observations suggest that CU06-1004 inhibits I/R-induced cerebral edema and astrocyte end-feet swelling by maintaining EC junction stability.

**Key messages:**

• CU06-1004 ameliorates I/R-induced cerebral injury.

• EC junction integrity was stabilized by CU06-1004 treatment after I/R.

• CU06-1004 reduces astrocyte end-feet swelling following I/R.

• EC junction stability affects astrocyte end-feet structure maintenance after I/R.

**Electronic supplementary material:**

The online version of this article (10.1007/s00109-020-01920-z) contains supplementary material, which is available to authorized users.

## Introduction

Cerebral ischemia, or stroke, has become a leading cause of death and disability, worldwide [1]. Ischemia/reperfusion (I/R) injury is a feature of stroke that occurs when the blood supply is restored after ischemia. Reperfusion can be achieved either through thrombolysis using thrombolytic reagents or the surgical removal of thrombi [2]. Despite known beneficial effects, reperfusion causes detrimental effects, including edema, blood‑brain barrier (BBB) breakdown, and astrocyte end-feet swelling in stroke patients [2–4]. However, the underlying mechanisms associated with reperfusion damage remain poorly understood. Therefore, methods to protect against stroke need to be developed to inhibit reperfusion-induced damage.

The dynamic activities of nervous tissue are regulated by blood flow in the brain, which is restricted by the BBB, whose structural components include endothelial cells (ECs), tight junctions, pericytes, and astrocyte end-feet [5]. ECs are connected by tight junction proteins, especially occludin, claudin-5, and zona occludense-1 (ZO-1), and are important for BBB permeability. Whereas, occludin and claudin-5 are transmembrane tight junction components, ZO-1 connects the tight junction to actin filaments [6]. The cortical actin ring is composed of cross-linked actin filaments that stabilize EC junctions [7]. Because EC junctions are disrupted during I/R, preventing the destruction of the cortical actin ring may be a promising therapeutic strategy for ameliorating I/R injury. Although ECs form the vessel walls, astrocyte end-feet closely surround capillaries through the vascular basement membrane, which is composed of extracellular matrix. Astrocyte adhesion regulates the quality of the BBB and can be disrupted by pathological conditions, such as stroke and neurological disorders [5]. Accordingly, investigations into stroke have further extended to include not only ECs but also astrocytes. Following I/R, astrocyte end-feet swelling is one of the earliest responses. Water channels and gap junctions have been reported to play important roles in the mediation of astrocyte end-feet swelling [3]. Aquaporins (AQPs) are bidirectional water channels that allow water diffusion, and AQP4 specifically localizes to astrocyte end-feet. Due to specialized features of end-feet, including a high density of orthogonal particle arrays containing AQP4, decreased polarity and increased expression of AQP4 cause edema following stroke [8]. Gap junctions consist of connexin (CX) proteins and allow the passage of molecules. ECs express CX37, 40, and 43, whereas, astrocytes express CX26, 30, and 43 [9]. Among them, CX43 is thought to be involved in the communication between ECs and astrocytes [10]. CX43 dysfunction leads to neuroinflammation, edema, and BBB disruption [9, 11]. However, the correlation between EC junction disruptions and astrocyte end-feet swelling following I/R remains unknown.

In previous studies, CU06-1004 (previously Sac-1004) enhanced the endothelial barrier, increasing cortical actin ring formation, through cAMP/Rac/cortical actin pathways, which prevented vascular endothelial growth factor-induced actin filament destabilization and reduced I/R-induced BBB breakdown in a rat I/R model [12, 13]. Because the mechanism of astrocyte end-feet disruption after I/R injury and whether the breakdown of EC junctions is closely involved in astrocyte end-feet disruption both remain elusive, here, we investigated the effects of CU06-1004 on edema and astrocyte end-feet swelling during I/R injury. Our data suggest that the EC junction stability is significantly correlated with the maintenance of proper astrocyte end-feet structure after cerebral I/R injury.

## Materials and methods

### Oral administration of CU06-1004

CU06-1004 was synthesized as previously described [12]. Briefly, CU06-1004 was synthesized via tetrahydropyran deprotection and subsequent glycosidation with 4,6-di-O-acetyl-2,3-didieoxyhex-2-enopyran, in the presence of an acid. A working solution of CU06-1004 was prepared in olive oil (O1514; MilliporeSigma, Burlington, MA, USA). Mice were fasted for 4 h before performing ischemia/reperfusion (I/R). Mice awakened from anesthesia 20 min after reperfusion and were then orally administered a single dose of 10 mg/kg CU06-1004 (CU06-1004-I/R group), in a volume of 10 mL/kg. Sham-operated (sham group) and vehicle-treated mice (vehicle-I/R group) were administered the same volume of suspension solution (olive oil, 10 mL/kg).

### Brain ischemia/reperfusion (I/R)

Mice were anesthetized by the intraperitoneal injection of tribromoethanol (Avertin, 2.5%). I/R was induced by middle cerebral artery (MCA) occlusion on the left side as previously described [13]. Briefly, the common carotid artery (CCA) was ligated at the distal side of its branching point. The external carotid artery (ECA) was dissected free and ligated at the distal side of the CCA branching point. A 6-0 monofilament suture (602156PK10; Doccol Corp, Sharon, MA, USA) was inserted into the ECA stump and gently advanced 8 mm into the internal carotid artery (ICA). The suture proceeded from the ICA to the MCA. After 1.5 h of ischemia, reperfusion was induced by the withdrawal of the suture. In the sham group, the same surgical procedures were performed without insertion of the suture.

### Oxygen-glucose deprivation/reoxygenation (OGD/R)

Human brain microvascular endothelial cells were purchased from Applied Cell Biology Research Institute (Kirkland, WA, USA). OGD/R was performed as previously described [14]. Confluent cells were washed and replaced with glucose-free Dulbecco’s Modified Eagle’s Medium (DMEM). Cells were then incubated with AnaeroPack (AN0025A; Thermo Fisher Scientific) for 6 h. After OGD, cells were reperfused by placing them in endothelial basal medium 2 (CC-3156; Lonza, Walkersville, MD, USA) containing vehicle or CU06-1004 (10 μg/mL) and were incubated in a 95% air/5% CO_2_ for 16 h. After 16 h, immunofluorescence staining was performed.

### Analysis of astrocyte end-feet diameter

Astrocyte end-feet diameter was measured as previously described [15]. After immunostaining of brain slices, the 3-dimensional images were captured using multiple XYZ imaging stacks, orthogonal to the plane. These images were imported into Image J, and the diameter was measured using ruler of X and Y axes.

### Isolation of mouse astrocytes

Astrocytes were isolated from four postnatal mice (1–2 days old) as previously described [16–18]. Gray cortices were minced, digested with 0.25% trypsin, and centrifuged. Pellets were resuspended in DMEM (HyClone, SH3024301; GE Healthcare Bio-Sciences, Pittsburgh, PA, USA) supplemented with 10% fetal bovine serum (HyClone, SH3008403), 100 U/mL penicillin, and 100 μg/mL streptomycin. Cells were seeded in T75 culture flasks pre-coated with 20 mg/mL poly-D-lysine. Culture media were changed every 2 days. After achieving confluency around 7–9 days in vitro, microglia were discarded from mixed glial cells via shaking on an orbital shaker at 180 rpm for 30 min. Cells were then washed and separated from oligodendrocyte precursor cells by shaking at 240 rpm for 6 h. The remaining adherent cells were > 98% positive for the astrocytic marker glial fibrillary acidic protein (GFAP).

### Animal studies

C57BL6/J male mice (8–10 weeks old) were purchased from RaonBio (Yongin, Korea). Five mice were used for each group. All mice were housed under controlled conditions (24 ± 1 °C, 12 h light/dark cycles, 55% humidity, and specific-pathogen-free) and provided with food and water ad libitum. All animal facilities and experiments were performed in accordance with the Korean Food and Drug Administration guidelines. All procedures were reviewed and approved by the Institutional Animal Care and Use Committee at Yonsei University (permit number IACUC-A-201904-892-02).

### Statistical analysis

Data are presented as the mean ± standard error of the mean (SEM). All statistical analyses were performed using Prism 5.0 (GraphPad Software; La Jolla, CA, USA). Data were statistically analyzed by one-way analysis of variance, with post-hoc Bonferroni’s multiple comparison tests, to determine ischemia-related differences among the experimental groups. To identify whether we had sufficient subjects to detect with statistics, a post-hoc power analysis was performed after the study using G-Power 3.1 software (https://download.cnet.com/s/g-power). Values of *P* < 0.05 were considered to be significant.

## Results

### CU06-1004 attenuates brain damage after I/R

**Fig. 1:**
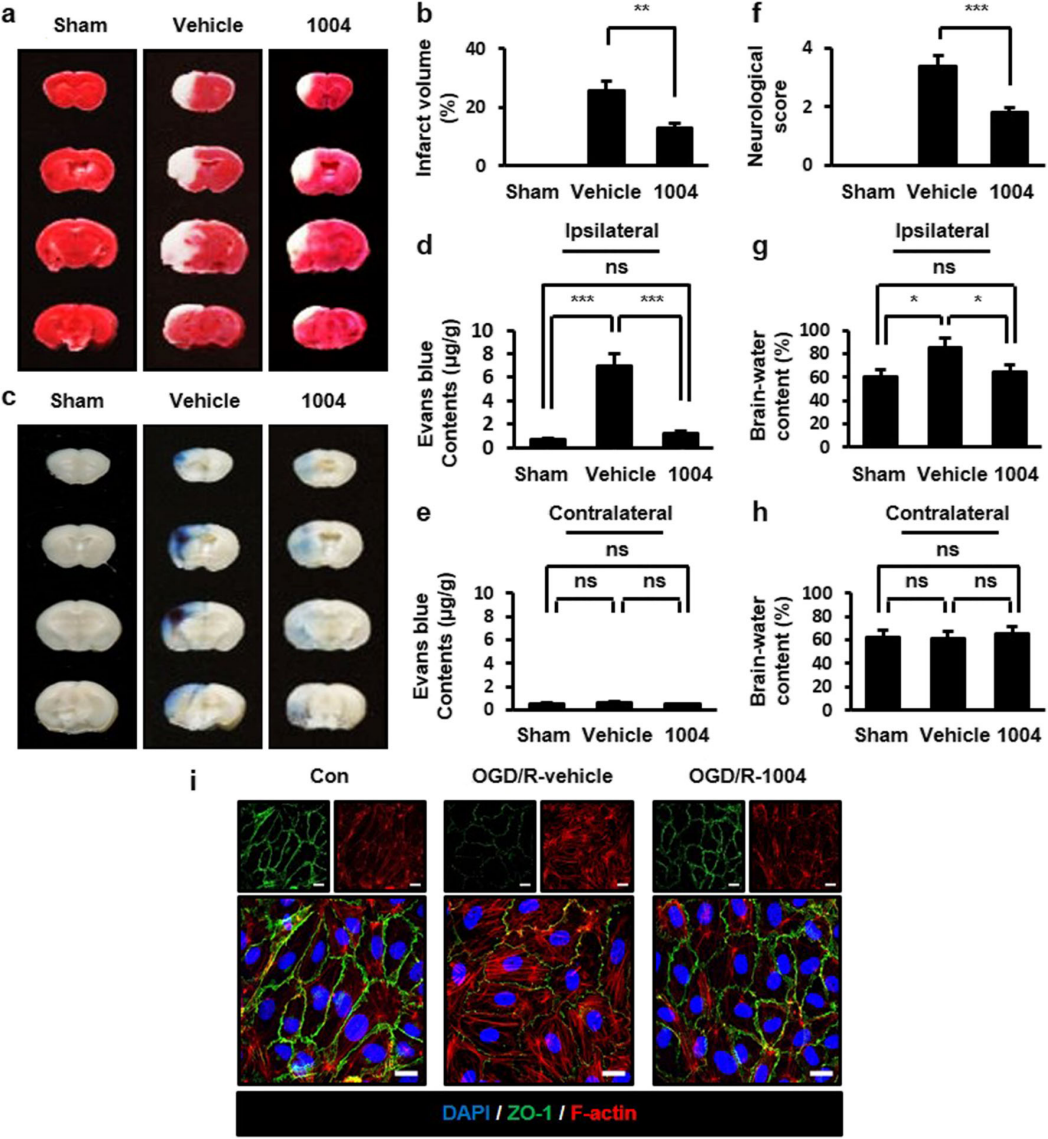
CU06-1004 attenuates brain injury and the disruption of EC junctions after I/R. Comparison of infarction volumes assessed by TTC staining in the sham, vehicle-I/R, and CU06-1004-I/R groups (**a**) and quantified (**b**). Evans blue extravasation from sham, vehicle-I/R, and CU06-1004-I/R groups (**c**) and the quantification of Evans blue in ipsilateral (**d**) and contralateral hemispheres (**e**). Neurological scores for sham, vehicle-I/R, and CU06-1004-I/R groups (**f**). Brain water content for sham, vehicle-I/R, and CU06–1004-I/R groups. Quantification of water content in the ipsilateral (**g**) and contralateral hemispheres (**h**). *n* = 5 per group. HBMECs were oxygen-glucose deprived for 6 h (**i**). After reoxygenation and treatment with vehicle or CU06-1004 (10 μg/mL) for 16 h, cells were stained with DAPI, ZO-1, and F-actin. Scale bars, 20 μm. *P < 0.05, **P < 0.01, and ***P < 0.001. Error bars indicate mean ± SEM. *ns*, not significant; *Con*, control; *OGD/R*, oxygen-glucose deprivation/reperfusion; *DAPI*, 4′,6-diamidino-2-phenylindole; *ZO-1*, zona occludens-1; *F-actin*, fibrous actin; *1004*, CU06-1004

A previous study evaluated the effects of the intravenous injection of CU06-1004 after I/R-induced brain damage [13]. To identify whether similar effects could be observed through other routes, we examined the oral administration of CU06-1004. Infarct volume was examined using 2,3,5-triphenyltetrazolium chloride staining. In the vehicle-I/R group, infarcted regions were observed compared with the sham group. However, in the CU06-1004-I/R group, fewer infarcted regions were observed compared with the vehicle-I/R group (Fig. 1a, b). The effects of CU06-1004 on blood‑brain barrier (BBB) permeability were evaluated with Evans blue (EB) extravasation. EB extravasation was increased in the vehicle-I/R group compared with the sham group. However, CU06-1004 significantly decreased this effect (Fig. 1c−e). Next, we measured neurological deficits. In the vehicle-I/R group, severe neurological deficits were exhibited compared with the sham group. However, CU06-1004 prevented neurological impairments induced by I/R (Fig. 1f). Brain edema was measured by the wet-dry method. In the vehicle-I/R group, brain water content was increased compared with the sham group. In the CU06-1004-I/R group, brain water content was reduced compared with the vehicle-I/R group (Fig. 1g, h). Taken together, these results indicate that the oral administration of CU06-1004 protects against I/R-induced cerebral injury.

### CU06-1004 inhibits EC junction disruption and enhances EC viability after oxygen-glucose deprivation/reoxygenation (OGD/R)

A prior study showed that the treatment of ECs with CU06-1004 inhibited cortical actin ring disruption following vascular endothelial growth factor stimulation [12]. To clarify the role of CU06-1004 in cortical actin ring maintenance, we performed OGD/R as an in vitro I/R model. Vehicle-treated human brain microvascular endothelial cells (HBMECs) showed the breakage of ZO-1 that connects tight junction-related proteins to actin filaments following OGD/R. However, CU06-1004 restored ZO-1, similar to the control group. Furthermore, the induction of stress fiber formation and decreased cortical actin ring formation, which were observed in the vehicle-treated group, were reversed by CU06-1004 treatment (Fig. 1i). HBMEC viability was also suppressed after OGD/R; however, CU06-1004 prevented this effect (Fig. S1a, b). These results demonstrate that CU06-1004 exerts protective effects in ECs by stabilizing tight junctions and the cortical actin ring after OGD/R.

### CU06-1004 suppresses astrocyte end-feet swelling and astrocyte activation after I/R

Previous evidence has shown that I/R-induced vascular junction disruption was inhibited by CU06-1004 [13]. Because pericyte attachment is also involved in vessel integrity stabilization, we examined pericyte coverage after I/R. Brains from the vehicle-I/R group showed reduced α-smooth muscle actin (α-SMA), pericyte marker, compared with the sham group. A superimposed lining of α-SMA and cluster of differentiation 31 (CD31), an EC marker, was observed in the CU06-1004-I/R group (Fig. S2a−e), indicating that CU06-1004 significantly blocks the disruption of the BBB structure after I/R injury.

**Fig. 2:**
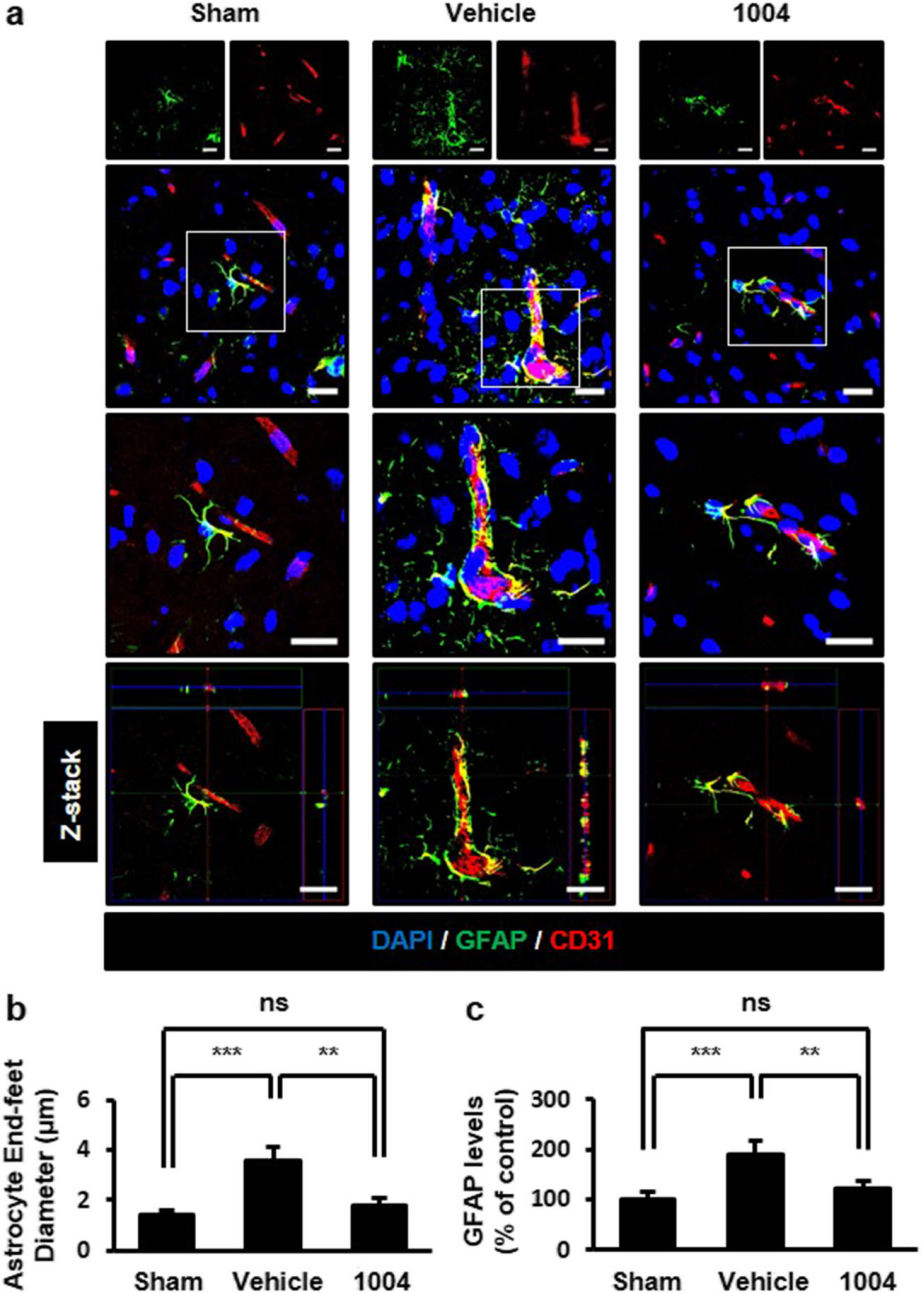
CU06-1004 inhibits astrocyte end-feet swelling and activation after I/R **a** Comparisons of astrocyte changes among the sham, vehicle-I/R, and CU06–1004-I/R groups. Square, enlarged image of the region. Tissues were immunostained for GFAP, as an astrocyte marker, and CD31, as an EC marker. **b** Quantification of astrocyte end-feet diameter and **c** GFAP expression levels. *n* = 5 per group. **P < 0.01 and ***P < 0.001. Scale bars, 20 μm. Error bars indicate mean ± SEM. *ns*, not significant; *DAPI*, 4′,6-diamidino-2-phenylindole; *GFAP*, glial fibrillary acidic protein; *CD31*, cluster of differentiation 31; *1004*, CU06-1004

Cerebral edema is one of the earliest responses to I/R due to astrocyte end-feet swelling [3]. End-feet swelling compresses vessels in the ischemic region, resulting in vascular hypoperfusion [19]. We determined end-feet swelling by measuring end-feet diameter in GFAP-labeled cells. Because astrocyte activation is induced by brain injury [3], GFAP levels were also measured. In the vehicle-I/R group, astrocyte abnormalities were evident with increased astrocyte numbers and diameters surrounding ECs. Astrocyte end-feet were swollen, losing fine contact with ECs. These astrocytic changes were significantly reversed by CU06-1004 (Fig. 2a−c).

To further investigate whether CU06-1004 directly affects astrocyte properties, we examined the viability and proliferation of primary astrocytes after CU06-1004 treatment. Isolated primary mouse astrocytes were immunostained for GFAP and CD11b (microglial marker) to confirm cellular identities before experimentation (Fig. S3a). Unlike the protective effects of CU06-1004 in ECs, CU06-1004 did not affect astrocyte properties with or without OGD/R (Fig. S3b−i). Collectively, these results suggest that CU06-1004 likely alleviates astrocyte end-feet destruction by stabilizing the vascular structure after I/R.

### CU06-1004 reduces increased aquaporin 4 (AQP4) levels after I/R

**Fig. 3:**
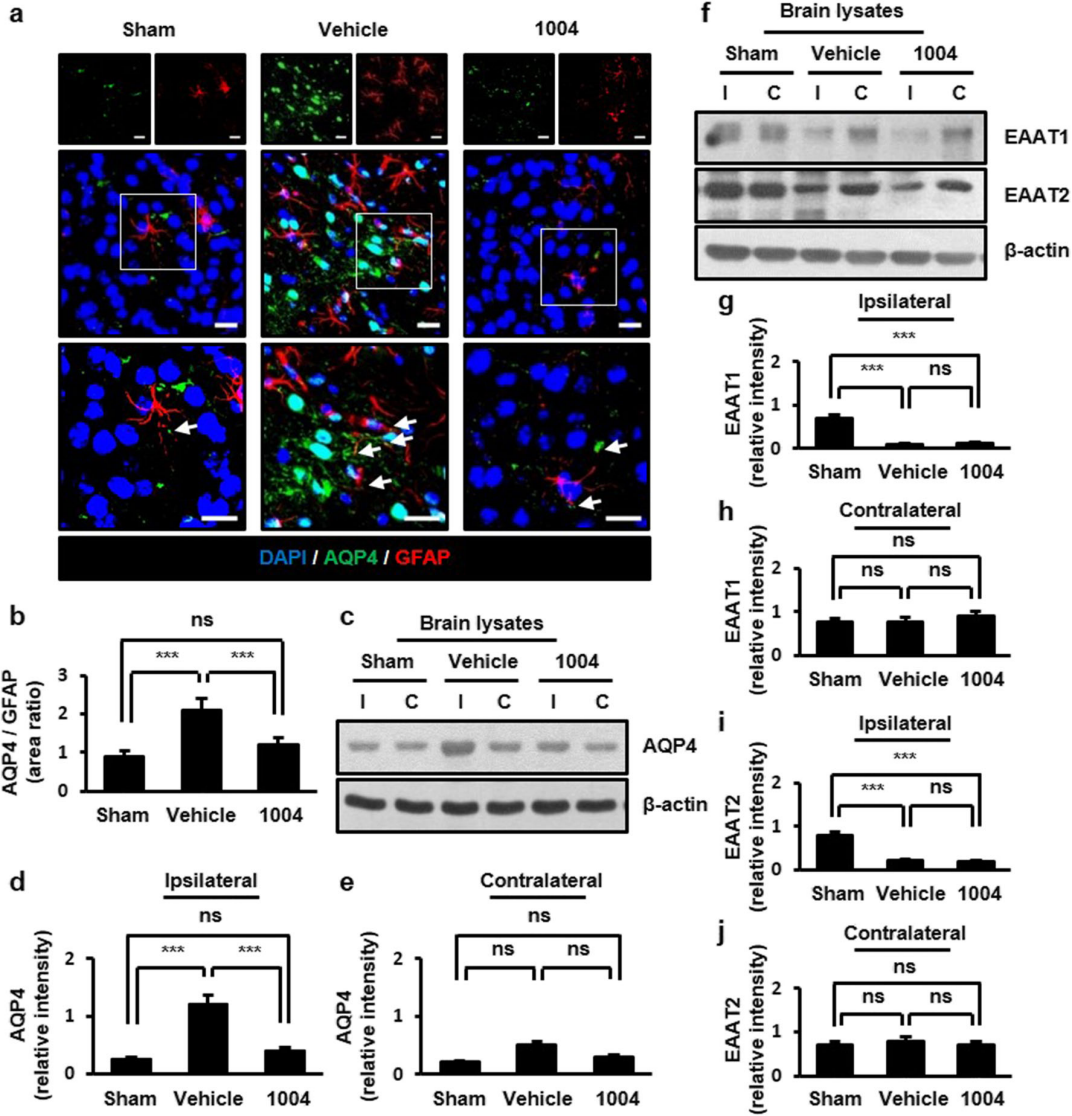
CU06-1004 reduces increased AQP4 expression after I/R. **a** Comparison of AQP4 expression in the sham, vehicle-I/R, and CU06-1004-I/R groups. Square: enlarged image of the region. Arrows show the presence of AQP4 on astrocytes. **b** Quantification of AQP4-positive astrocytes. *n* = 5 per group; scale bars:, 20 μm. **c** Effects of CU06-1004 administration on AQP4 expression in brain lysates. **d, e** Quantification of blots using ImageJ software. *n* = 3 independent experiments. **f** Effects of CU06-1004 administration on EAAT1/2 expression in brain lysates. **g**−**j** Quantification of blots using ImageJ software. *n* = 3 independent experiments. ***P < 0.001. Error bars indicate mean ± SEM. *ns*, not significant; *DAPI*, 4′,6-diamidino-2-phenylindole; *AQP4*, aquaporin 4; *GFAP*, glial fibrillary acidic protein; *I*, ipsilateral hemisphere; *C*, contralateral hemisphere; *1004*, CU06-1004

AQP4 is highly expressed on astrocyte end-feet and regulates water transport between blood and brain tissue [3]. AQP4 is up-regulated by cerebral ischemic injury, especially in astrocyte end-feet adjacent to blood vessels [20, 21]. In the sham group, AQP4 was mainly expressed in astrocyte end-feet. In the vehicle-I/R group, AQP4 was not only localized to astrocyte end-feet but also distributed along astrocytic processes. CU06-1004 administration resulted in AQP4 distribution similar to that in the sham group (Fig. 3a, b). Similar results were also observed in ECs (Fig. S4a, b). We further confirmed AQP4 expression by western blotting. In the ipsilateral hemispheres of the vehicle-I/R group, AQP4 levels were increased compared with those in both the sham group and the corresponding contralateral hemispheres. CU06-1004 significantly reduced AQP4 expression to the sham group in the ipsilateral hemispheres (Fig. 3c−e).

Astrocyte end-feet swelling can be caused by the increased uptake of excitatory amino acid neurotransmitters, such as glutamate. Glutamate is cleared by excitatory amino acid transporters 1/2 (EAAT1/2) in astrocyte end-feet surrounding neurons [22]. Reduced EAAT1/2 expressions have been reported in cerebral ischemia [23, 24]. In the vehicle-I/R group, ipsilateral hemispheres showed greatly reduced EAAT1/2 expression as previously reported. However, CU06-1004 failed to increase the EAAT1/2 expression (Fig. 3f−j). Taken together, these observations suggest that the effect of CU06-1004 on astrocyte end-feet swelling is likely due to the regulation of AQP4 but not EAAT1/2 in astrocytes.

### CU06-1004 alleviates increased connexin (CX) 43 levels after I/R

**Fig. 4:**
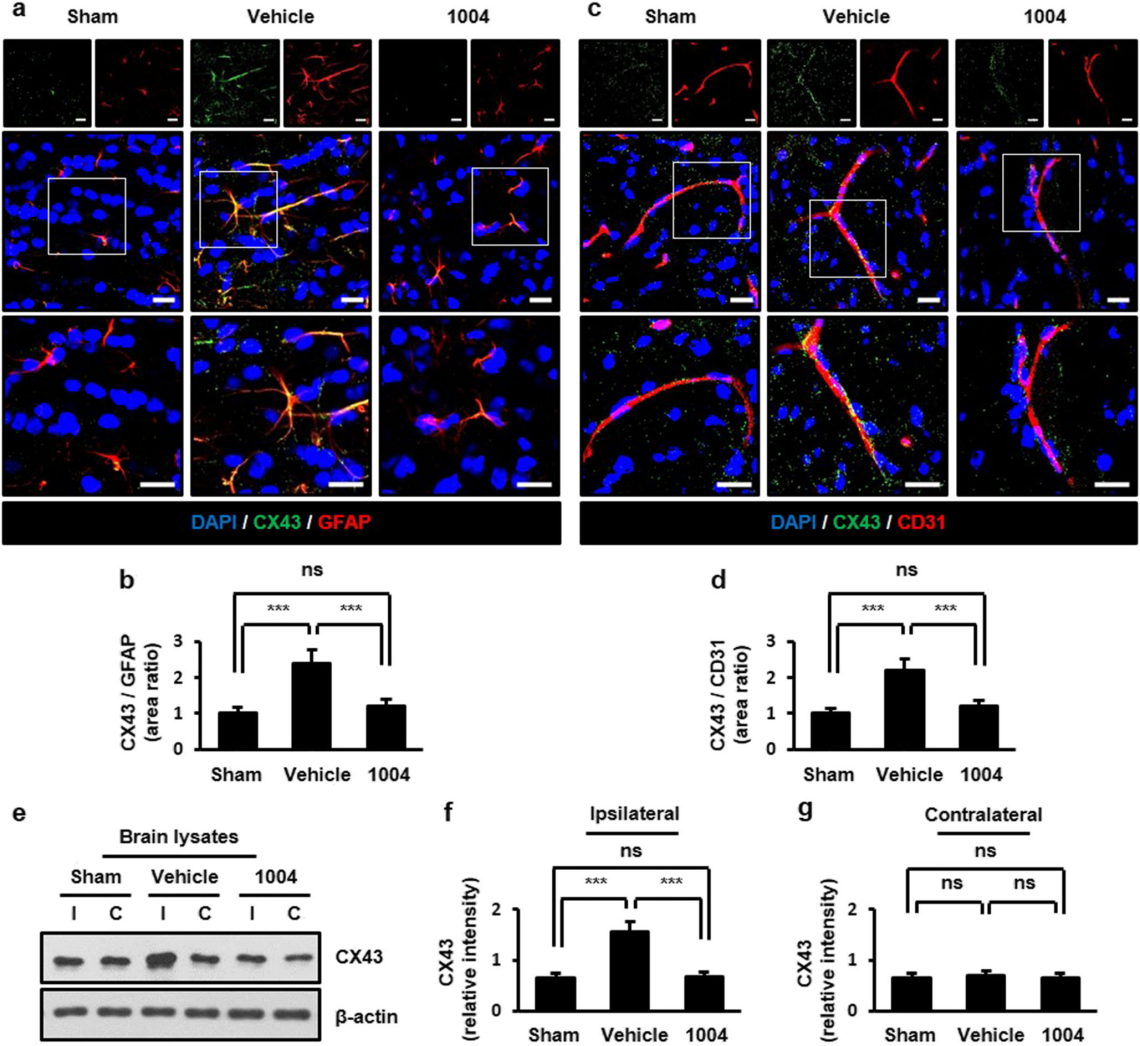
CU06-1004 alleviates increased CX43 expression after I/R. Comparison of CX43 expression in the sham, vehicle-I/R, and CU06-1004-I/R groups (**a**, **c**). Square, enlarged image of the region. Quantification of CX43-positive astrocytes (**b**) and blood vessels (**d**). *n* = 5 per group; scale bars, 20 μm. Effects of CU06–1004 administration on CX43 expression in brain lysates (**e**). Quantification of blots using ImageJ software (**f**, **g**). *n* = 3 independent experiments. ***P < 0.001. Error bars indicate mean ± SEM. *ns*, not significant; *DAPI*, 4′,6-diamidino-2-phenylindole; *CX43*, connexin 43; *GFAP*, glial fibrillary acidic protein; *CD31*, cluster of differentiation 31; *I*, ipsilateral hemisphere; *C*, contralateral hemisphere; *1004*, CU06-1004

Cell adhesion molecules, known as CXs, also mediate astrocyte end-feet swelling. Among the CX family, CX43 is expressed in both ECs and astrocytes [25], and astrocytic CX43 levels are increased after I/R injury [26, 27]. In the vehicle-I/R group, CX43-positive astrocytes and ECs were increased compared with the sham group. These changes were significantly reversed by CU06-1004 (Fig. 4a−d). We further confirmed the inhibitory effects of CU06-1004 on CX43 expression by western blotting (Fig. 4e−g).

### CU06-1004 inhibits degradation of basal membrane and extracellular matrix (ECM) proteins after I/R

**Fig. 5:**
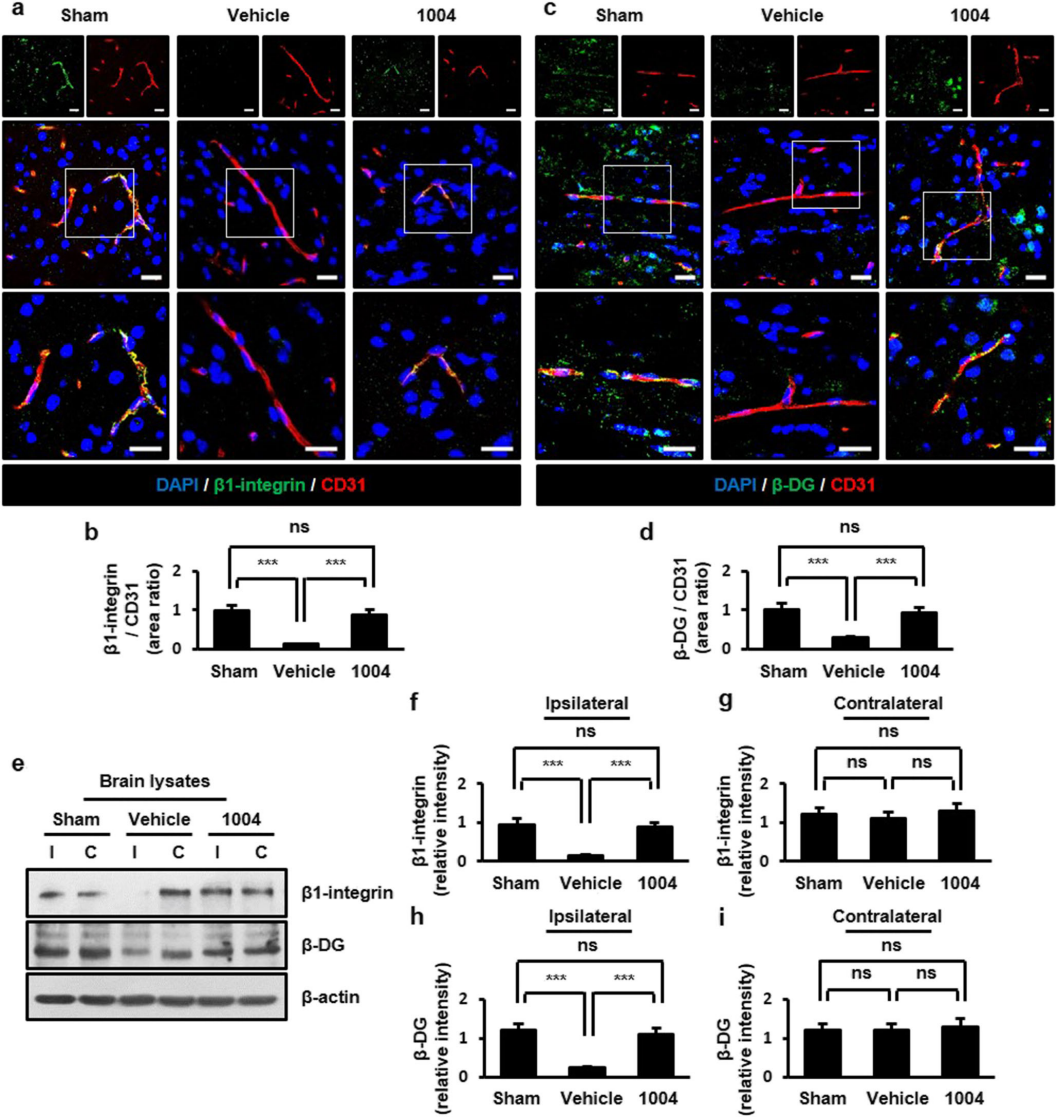
CU06-1004 prevents the disruption of basal membrane proteins in ECs after I/R. Comparison of β1-integrin (**a**) and β-DG (**c**) expression in the sham, vehicle-I/R, and CU06-1004-I/R groups. Square, enlarged image of the region. Quantification of β1-integrin- (**b**) and β-DG- positive (**d**) blood vessels. *n* = 5 per group; scale bars, 20 μm. Effects of CU06–1004 administration on β1-integrin and β-DG expression in brain lysates (**e**). Quantification of blots using ImageJ software (**f**−**i**). *n* = 3 independent experiments. ***P < 0.001. Error bars indicate mean ± SEM. *ns*, not significant; *DAPI*, 4′,6-diamidino-2-phenylindole; *DG*, dystroglycan; *CD31*, cluster of differentiation 31; *I*, ipsilateral hemisphere; *C*, contralateral hemisphere; *1004*, CU06-1004

**Fig. 6:**
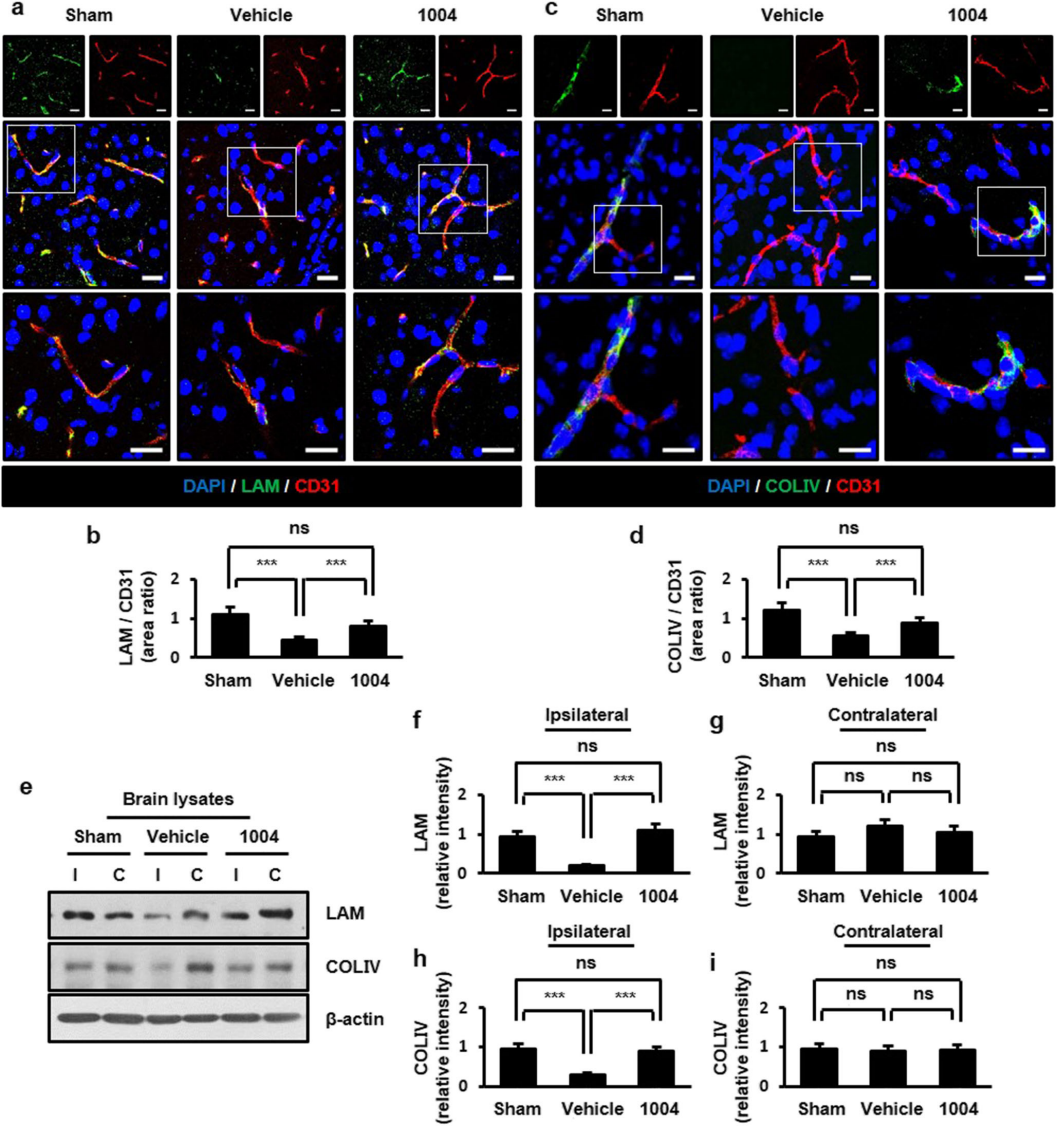
CU06-1004 inhibits the reduction in extracellular matrix proteins after I/R. Comparison of LAM (**a**) and COLIV (**c**) expression in the sham, vehicle-I/R, and CU06-1004-I/R groups. Square, enlarged image of the region. Quantification of LAM- (**b**) and COLIV-positive (**d**) blood vessels. *n* = 5 per group; scale bars, 20 μm. Effects of CU06-1004 administration on LAM and COLIV expression in brain lysates (**e**). Quantification of blots using ImageJ software (**f**−**i**). *n* = 3 independent experiments. ***P < 0.001. Error bars indicate mean ± SEM. *ns*, not significant; *DAPI*, 4′,6-diamidino-2-phenylindole; *LAM*, laminin; *COLIV*, collagen type IV; *CD31*, cluster of differentiation 31; *I*, ipsilateral hemisphere; *C*, contralateral hemisphere; *1004*, CU06-1004

The cortical actin ring binds to basal membrane proteins of EC, such as β1-integrin and β-dystroglycan (β-DG). Basal membrane proteins of EC directly anchor to the ECM. Astrocyte end-feet also anchor to ECs through the ECM [28, 29]. Because laminin (LAM), an ECM component in ECs, is important for the localization and expression of AQP4 at astrocyte end-feet [30], we further analyzed whether CU06-1004 affects the expression of basal membrane and ECM proteins after I/R injury. In the vehicle-I/R group, β1-integrin- and β-DG-positive ECs were decreased compared with the sham group. However, CU06-1004 significantly increased β1-integrin- and β-DG-positive ECs compared with the vehicle-I/R group (Fig. 5a−d). A similar result was observed in astrocytes (Fig. S5a−d). Western blotting further confirmed that CU06-1004 inhibited the I/R-induced degradation of these proteins in the ipsilateral hemispheres (Fig. 5e−i). Similarly, the expressions of LAM and collagen type IV (COLIV), which are major components of the ECM, were decreased in the vehicle-I/R group compared with the sham group. CU06-1004 significantly returned LAM and COLIV expression similar to those in the sham group (Fig. 6a−d). These protein levels in brain tissues were further confirmed by western blotting (Fig. 6e−i). Taken together, our results suggest that CU06-1004 inhibits loss of basal membrane and ECM proteins at the BBB, which could contribute to the rescue of astrocyte end-feet structure after I/R injury.

## Discussion

Cerebral ischemia, or stroke, is a common cerebrovascular disease with high morbidity and disability [2]. I/R injury refers to the recovery of blood flow after stroke, which is accompanied by BBB disruptions, oxidative stress injury, and inflammation [31]. BBB disruptions cause edema and hemorrhage, resulting in irreversible brain tissue damage [32]. Thus, the maintenance of BBB integrity may be a key treatment strategy for preventing I/R injury. In previous studies, CU06-1004 was used as a vascular leakage blocker to suppress vascular endothelial growth factor-induced permeability and improved endothelial junctions during I/R injury and tumor angiogenesis [13, 33]. Interestingly, the oral administration of CU06-1004 following I/R significantly suppressed I/R-induced infarct, vascular leakage, behavioral impairment, and astrocyte activation. A single administration of CU06-1004, immediately after onset of reperfusion, inhibited cerebral I/R-induced damage. CU06-1004 stabilizes tight junction-related proteins and the cortical actin ring in ECs [12]. Here, we found that CU06-1004 suppressed OGD/R-induced EC junction disruptions. Therefore, CU06-1004 has a strong capacity to protect ECs from damage and to maintain intact vasculature against I/R injury. In addition to effects on EC junctions, CU06-1004 also preserved intact astrocyte end-feet following I/R. Because CU06-1004 did not directly affect astrocyte viability and proliferation, these effects on astrocyte end-feet maintenance are intriguing. Under normal conditions, astrocyte end-feet closely localize to ECs through interactions between basal membrane and ECM proteins [3]. After I/R injury, the disruption of both EC junctions and astrocytes appeared and was sustained. These abnormalities were reversed by CU06-1004 treatment. Thus, the effects of CU06-1004 on astrocyte end-feet structure are likely mediated by primary effects on EC junctions. Collectively, the direct and indirect effects of CU06-1004 on ECs and astrocytes were able to protect the BBB structure from I/R injury and subsequently reduce edema, astrocyte activation, and inflammation, resulting in the alleviation of neuronal damage. Taken together, these results suggest that the protection of EC junctions is important for the maintenance of astrocyte end-feet and the control of astrocyte activation.

The outermost layer of the BBB is a tightly woven mesh of astrocyte end-feet. Astrocytes are morphologically polarized cells, and nearly every astrocyte extends at least one end-foot that contacts a vessel. Thus, BBB dysfunction is associated with the loss of astrocyte polarity [34]. The end-foot membrane domain is enriched in channels used for water and ion movements [35]. The expression of these channels are increased following stroke, and AQP4, EAAT 1/2, and CX43 have been reported to play important roles in the mediation of astrocyte end-feet swelling [8]. AQP4 is highly expressed on astrocyte end-feet and regulates water transportation. Because AQP4 is normally arranged in a polar manner, the increased mispolarization and expression of AQP4 cause neurological disorders [34]. AQP4 knockout mice show reduced cerebral infarction and edema following ischemia and I/R induction [36, 37]. Increased AQP4 expression following I/R injury was inhibited by CU06-1004, suggesting that CU06-1004 may suppress AQP4-mediated changes in astrocyte volume. Excessive extracellular glutamate enters astrocyte end-feet through EAAT1/2 after neurotransmission. Increased EAAT1/2 expression reduces infarction volume and improves behavior following I/R injury [38, 39]. Vehicle-treated brains showed reduced EAAT1/2 expression; however, CU06-1004 did not alter EAAT1/2 expression following I/R. Because EAATs are mainly expressed in astrocyte end-feet surrounding neurons, CU06-1004 is likely to act on astrocyte end-feet surrounding ECs. Astrocyte end-feet and ECs are interconnected by gap junctions, which allow the non-selective movement of small molecules. Cerebral I/R and OGD/R injuries increase CX43 expression and exacerbate neuroinflammation [40]. CX43 expression was increased after I/R injury, which was inhibited by CU06-1004. Collectively, these results suggest that CU06-1004 inhibits increased AQP4 and CX43 expression without affecting neurotransmission, and this activity contributes to the attenuation of astrocyte end-feet swelling after I/R injury.

The BM is located on the abluminal side of ECs [41]. In the brain, sandwiched between ECs and astrocyte end-feet contributes to BM formation by synthesizing and depositing ECM proteins, such as LAM and COLIV, suggesting that BM participates in BBB maintenance and vascular integrity [42]. ECM proteins can exert these functions by binding receptors, such as integrin and DG, on ECs and astrocytes [43]. According to Sato et al. and Rurak et al., DG knockdown or the impairment of DG binding to LAM causes the delocalization and reduced expression of AQP4 in astrocyte process formations [44, 45]. *Lamα2* knockout mice showed increased cerebral edema and reduced BBB integrity and basal membrane protein expression, such as β1-integrin and β-DG [46, 47]. Based on these reports, we postulated that AQP4 and CX43 expression may be modulated by ECM and basal membrane proteins. CU06-1004 suppressed the degradation of β1-integrin, β-DG, LAM, and COLIV following I/R. Collectively, when the cortical actin ring in ECs becomes unstable after I/R, basal membrane proteins that bind to the cortical actin ring become disrupted, resulting in the degradation of ECM proteins that anchor basal membrane proteins. These activities cause instability of junctions between ECs and astrocyte end-feet. AQP4 expression is also increased when the basal membrane proteins in the astrocyte end-feet become degraded [44, 45]. Therefore, when the basal membrane and ECM proteins in ECs become stabilized, the increased AQP4 expression induced by I/R becomes suppressed. In conclusion, stabilizing EC junctions by CU06-1004 can attenuate excessive water movement through AQP4 and contribute to protection of astrocyte-EC interactions. (Fig. S6a, b).

Current treatments for stroke are largely dependent on thrombolytic reagents-mediated thrombolysis. However, this strategy cannot protect against reperfusion-induced injury, which is a critical challenge during stroke treatment [48]. CU06-1004 was previously reported to alleviate reperfusion-induced injury by enhancing EC junctions [13]. Here, we evidently showed that the reperfusion-induced disruptions of EC junctions and astrocyte end-feet were significantly correlated. However, early administrations of CU06-1004 have limitation for treatment of I/R, which may diminish clinical relevance. Therefore, further research is needed to elucidate whether CU06-1004 can be exerted positive effects in clinical conditions, such as long term-induced I/R.

## Electronic supplementary material

ESM 1 (PDF 763 kb)

ESM 2 (PDF 274 kb)

## Abbreviations

α-SMA: α-smooth muscle actin
AQP: Aquaporin
BBB: Blood-brain barrier
BM: Basement membrane
CD31: Cluster of differentiation 31
COLIV: Collagen type IV
CX: Connexin
DG: Dystroglycan
EAAT: Excitatory amino acid transporter
EB: Evans blue
EC: Endothelial cell
ECM: Extracellular matrix
GFAP: Glial fibrillary acidic protein
HBMEC: Human brain microvascular endothelial cell
I/R: Ischemia/reperfusion
LAM: Laminin
OGD/R: Oxygen-glucose deprivation/reoxygenation
TTC: 2,3,5-triphenyltetrazolium chloride
ZO-1: Zona occludens-1
