# Supplementary material for: CU06-1004 (endothelial dysfunction blocker) ameliorates astrocyte end-feet swelling by stabilizing endothelial cell junctions in cerebral ischemia/reperfusion injury
Source: J Mol Med (Berl). 2020 May 16;98(6):875–86. doi: 10.1007/s00109-020-01920-z (PMC7297708; doi:10.1007/s00109-020-01920-z)
Supplement: Supplementary file 1 — (PDF 763 kb) [file 109_2020_1920_MOESM1_ESM.pdf]

The endothelial dysfunction blocker CU06-1004 ameliorates astrocyte end-feet swelling by stabilizing endothelial cell junctions in cerebral ischemia/reperfusion injury

Dong Young Kim[1], Haiying Zhang[1, 2], Songyi Park[1], Yeaji Kim[1], Cho-Rong Bae[1], Young-Myeong Kim[3], Young-Guen Kwon[1]

[1]Department of Biochemistry, College of Life science and Biotechnology, Yonsei University, Seoul, Republic of Korea
[2]CURACLE Co., Ltd., Seongnam-si, Gyeonggi-do, Republic of Korea
[3]Department of Molecular and Cellular Biochemistry, School of Medicine, Kangwon National University, Chuncheon-si, Gangwon-do, Republic of Korea

Corresponding author:
Young-Guen Kwon, Ph.D.
Department of Biochemistry, College of Life Science and Biotechnology, Yonsei University,
50 Yonsei-ro, Seodaemun-gu, Seoul, 03722, Republic of Korea
Tel: 82-2-2123-2903; Fax: 82-2-362-9897
E-mail: ygkwon@yonsei.ac.kr

Supplementary Materials and Methods

Neurological scores

Neurological defects were evaluated after 16 h reperfusion as previously described [1]. The scores were as follows: 0, no observable neurological deficit; 1, flexion of contralateral torso and forelimbs upon lifting the whole animal by the tail; 2, circling to the contralateral side when held by the tail with feet on the floor; 3, spontaneous circling to the contralateral side; 4, no spontaneous motor activity. For consistency, scoring was repeated three times.

Measurement of infarction volume

Infarction volume was measured as previously described [1]. Briefly, after 16 h reperfusion, mice were anesthetized by intraperitoneal injection of 2.5% Avertin. Brains were harvested and cut into 2 mm coronal slices using a brain matrix. Each slice was incubated with 2% 2,3,5-triphenyltetrazolium chloride (TTC) at 37℃ for 20 min. After the TTC reaction, infarction was marked by unstained areas, whereas normal areas were stained red. Infarction was quantified using Image J software (National Institutes of Health, Bethesda, MD, USA). To eliminate the effects of edema in the ipsilateral hemisphere, the infarction volume was calculated as follows: [(contralateral hemisphere – undamaged ipsilateral hemisphere)/(contralateral hemisphere × 2) × 100] %.

Determination of blood-brain barrier (BBB) permeability and brain-water content

BBB permeability was assessed by Evans blue (EB) extravasation using a previously described method with slight modification [1]. Briefly, after 16 h reperfusion, EB (2% in saline, 4 mL/kg) was injected into the tail vein. Mice were anesthetized by intraperitoneal injection of 2.5% Avertin 3 h after injection. Brains were harvested, and each hemisphere was weighed, homogenized in PBS, and centrifuged at 2,000 g for 15 min at 4℃. The resulting supernatant was added to an equal volume of trichloroacetic acid. After overnight incubation and centrifugation at 2,000 g for 15 min at 4℃, absorbance of the supernatant was measured by spectrophotometer at 620 nm. The dye content in the brain was calculated against a standard curve and expressed as μg/g. Brain-water content was calculated using the following formula: [(wet weight – dry weight)/wet weight × 100] %. After 16 h reperfusion, ipsilateral and contralateral hemispheres were weighed before (wet weight) and after drying at 80℃ overnight (dry weight).

Immunofluorescence staining of brain sections

Brains were fixed in 4% paraformaldehyde solution (pH 7.4) overnight at 4℃. Brains were then dehydrated in 15% sucrose solution overnight at 4℃ and transferred to 30% sucrose solution at 4℃

until the brains completely sank. Fixed brains were embedded in optimal cutting temperature (OCT) compound (FSC22 clear; Leica Biosystems, Wetzlar, Germany) for 30 min at room temperature, transferred to an embedding mold filled with OCT, and frozen on dry ice. Frozen brains were cut to a 20 μm thickness at −20°C (CM1850; Leica Biosystems), and sections were immunostained on slides. The sections were fixed in acetone at −80°C for 30 min and air dried. OCT compound was removed with running water. The sections were incubated in blocking solution (X0909; DAKO, Carpinteria, CA, USA) for 1 h at room temperature and incubated overnight with anti-mouse CD31 (550274, 1:100; BD Biosciences, San Jose, CA, USA), GFAP (MAB360, 1:100; MilliporeSigma, Burlington, MA, USA), α-SMA (ab5694, 1:100; Abcam, Cambridge, MA, USA), AQP4 (ab46182, 1:100; Abcam), CX43 (#3512, 1:100; Cell Signaling Technology, Danvers, MA, USA), β1-integrin (ab183666, 1:100; Abcam), β-dystroglycan (PA5-34908, 1:100; Thermo Fischer Scientific, Waltham, MA, USA), laminin (PA1-16730, 1:100; Thermo Fischer Scientific), or collagen type IV (ab6586, 1:100; Abcam) antibodies at 4℃. Sections were then washed with 0.1% Triton X-100 in PBS, incubated with anti-rat Alexa Fluor 594 (A21209), anti-mouse Alexa Fluor 488 (A11001), anti-mouse Alexa Fluor 594 (A11005), anti-rabbit Alexa Fluor 488 (A21206, 1:200; Thermo Fisher Scientific), or DAPI (D8417; 1 μg/mL, MilliporeSigma) overnight at 4℃, and then washed with 0.1% Triton X-100 in PBS. All antibodies were dissolved in antibody diluent (S3022, DAKO). Sections were photographed with a confocal microscope (LSM 700 META; Carl Zeiss, Oberkochen, Germany).

Western blot analysis

Brains were lysed in cell lysis buffer (100 mM Tris-Cl, pH 7.4, 5 mM EDTA, 50 mM NaCl, 0.5% NP-40, 1% Triton X-100) containing a protease inhibitor cocktail (05892970001; Roche, Mannheim, Germany). Lysates were centrifuged at 14,000 rpm for 15 min, and supernatants were collected. Proteins were separated by 8 or 10% sodium dodecyl sulfate polyacrylamide gel electrophoresis (SDS-PAGE) and then transferred to polyvinylidene difluoride membranes (IPVH00010, MilliporeSigma).

Membranes were incubated with primary antibodies (1:1,000) overnight at 4℃ and with secondary antibodies (1:3,000) for 45 min at room temperature. The primary antibodies against α-SMA, AQP4, CX43, β1-integrin, β-dystroglycan, laminin, or collagen type IV were the same used in the staining above. EAAT1 (ab416) and EAAT2 (ab41621) antibodies were purchased from Abcam. β-actin (MA5-15739) was purchased from Thermo Fisher Scientific. Goat anti-rabbit-HRP (31460) and goat anti-mouse-HRP (31430; Thermo Fisher Scientific) were used as the secondary antibodies. Detection was performed using ECL (32106; Thermo Fisher Scientific) according to the manufacturer's instructions.

MTT assay

Cell survival was analyzed by MTT assay. Cells were seeded at a density of 1 × 10^4 cells/mL. ECs and astrocytes were seeded in 2% gelatin- and poly-D-lysine-coated 96-well plates, respectively, and incubated overnight. Cells were then switched to serum-free media and treated with vehicle or CU06-1004 after OGD/R. After 48 h, 3-(4, 5-Dimethyl-2-thiazolyl)-2, 5-diphenyl-2H-tetrazolium bromide (MTT) (0.1 mg/mL) was added, and cells were incubated at 37℃ for 3 h. Residual MTT was removed, and the crystals were dissolved by incubating with dimethyl sulfoxide:ethanol (1:1). The absorbance at 560 nm was measured using a spectrophotometer.

5-bromo-2-deoxyuridine (BrdU) assay

Astrocytes proliferation was analyzed by BrdU assay. Astrocytes were seeded at a density of 7 × 10^4 cells/mL in 35-mm plates. After OGD/R, cells were treated with vehicle or CU06-1004 and labeled with 10 μM of BrdU for 16 h. The cells were then incubated with 2 M HCl to hydrolyze DNA for 30 min at room temperature. The cells were washed with PBS and immunofluorescently stained with a BrdU antibody.

Immunofluorescence staining of cells

Astrocytes were fixed in 4% paraformaldehyde (pH 7.4) for 20 min at room temperature and permeabilized with 0.1% Triton X-100 in PBS at 4℃. Cells were incubated with GFAP (1:300), CD11b (550282, 1:300; BD Biosciences), BrdU (sc-32323, 1:300; Santa Cruz Biotechnology, Inc., Dallas, Texas, USA), or ZO-1 (617300, 1:300; Thermo Fisher Scientific) overnight at 4℃. Cells were then rinsed with PBS and incubated with anti-mouse Alexa Fluor 488 (1:600), anti-rat Alexa Fluor 594 (1:600), or anti-rabbit Alexa Fluor 488 (1:600) antibodies for 60 min at room temperature. Next, cells were labeled with DAPI (1 μg/mL) or F-actin (R415, 1:300; Thermo Fisher Scientific) for 30 min at room temperature. Samples were analyzed using a confocal microscope.

Reference

1. Zhang H, Park JH, Maharjan S, Park JA, Choi KS, Park H, Jeong Y, Ahn JH, Kim IH, Lee JC et al (2017) Sac-1004, a vascular leakage blocker, reduces cerebral ischemia-reperfusion injury by suppressing blood–brain barrier disruption and inflammation. J Neuroinflammation 14:122. https://doi.org/10.1186/s12974-017-0897-3

Supplementary Figures

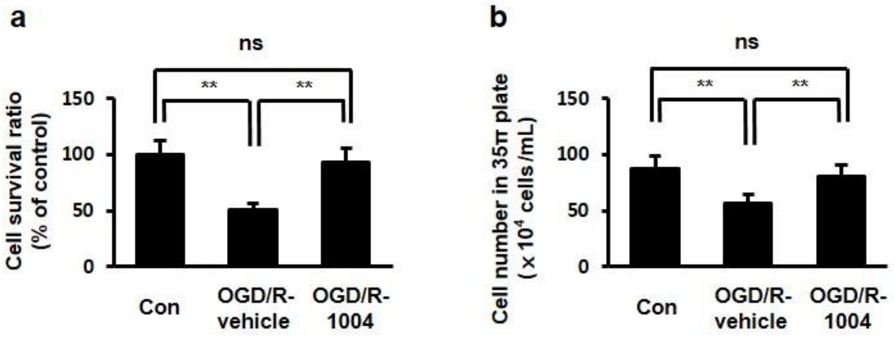

Fig. S1 CU06-1004 protects HBMEC viability after OGD/R.

Viability was measured by MTT assay (a) and cell counting (b). **a, b** HBMECs were oxygen-glucose deprived for 6 h. After reoxygenation and treatment with vehicle or CU06-1004 (10 μg/mL) for 16 h, cells were added MTT for 48 h (a) or counted (b). $^{**}P < 0.01$. Error bars indicate mean ± SEM. ns, not significant; Con, control; OGD/R, oxygen glucose deprivation/reperfusion; 1004, CU06-1004

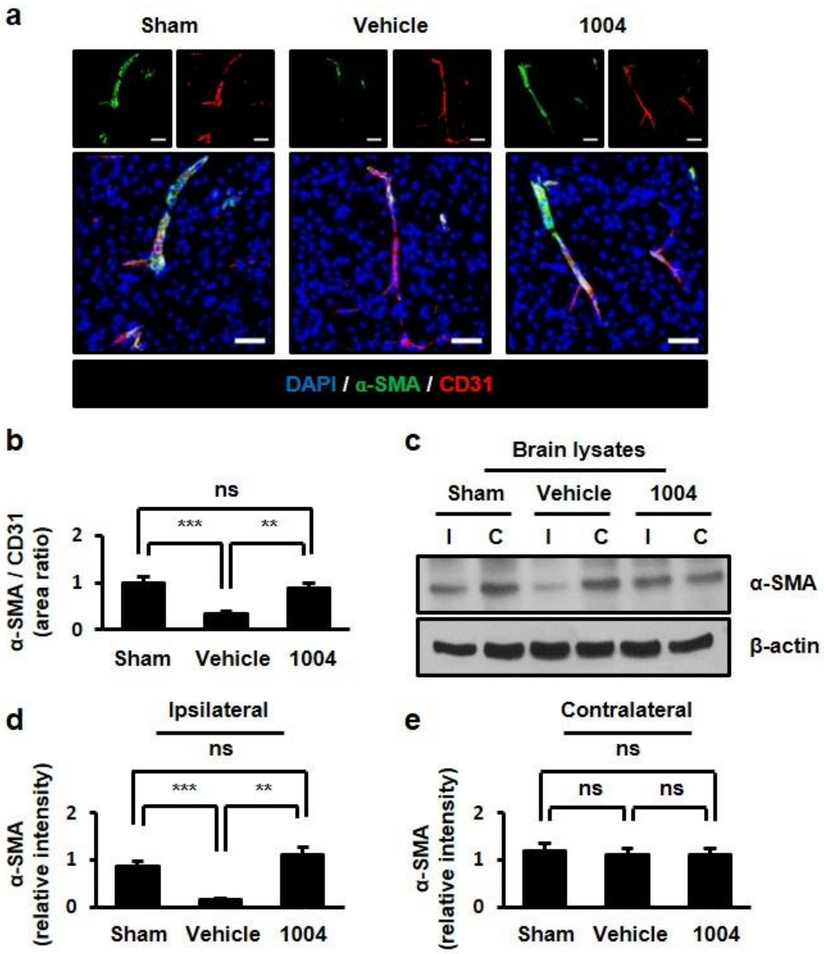

Fig. S2 CU06-1004 prevents pericyte disruption after I/R.

a Comparison of α-SMA expression in the sham, vehicle-I/R, and CU06-1004-I/R groups. **b** Quantification of α-SMA-positive blood vessels. n = 5 per group; scale bars: 50 μm. **c** Effects of CU06-1004 administration on α-SMA expression in brain lysates. **d, e** Quantification of blots using image J software. n = 3 independent experiments. **P < 0.01 and ***P < 0.001. Error bars indicate mean ± SEM. ns, not significant; DAPI, 4′,6-diamidino-2-phenylindole; α-SMA, α-smooth muscle actin; CD31, cluster of differentiation 31; I, ipsilateral hemisphere; C, contralateral hemisphere; 1004, CU06-1004

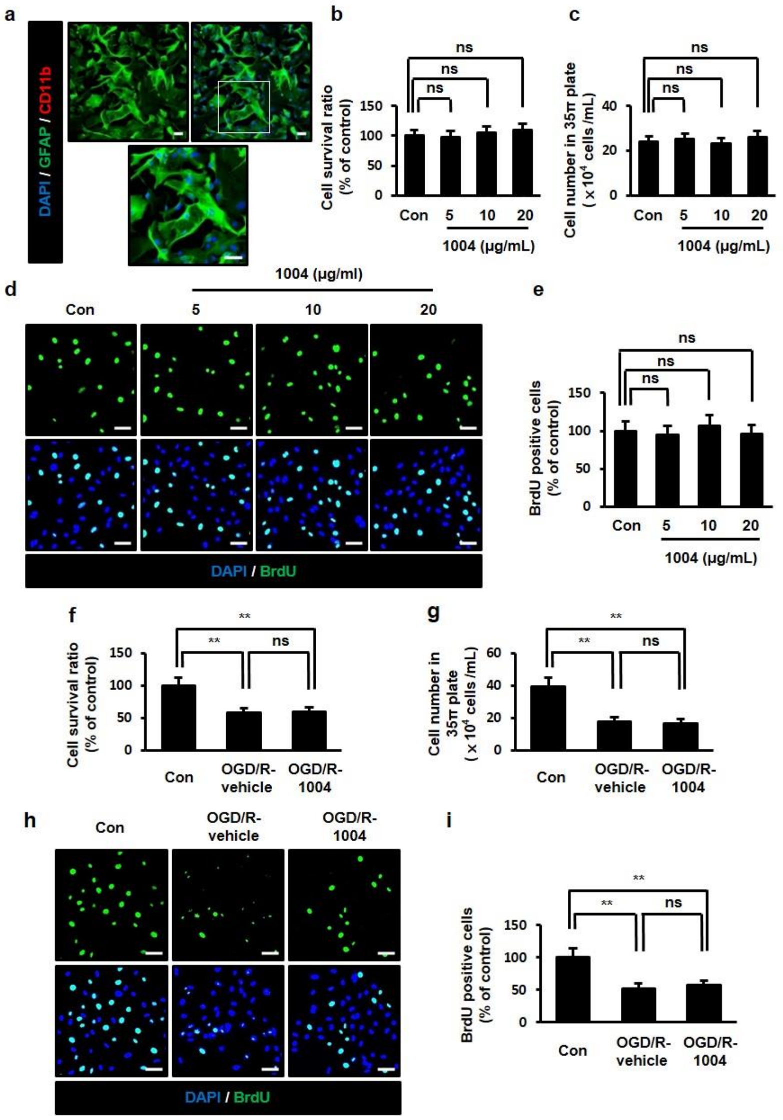

Fig. S3 CU06-1004 did not directly affect astrocyte viability and proliferation with or without OGD/R. **a** Astrocytes were isolated from mouse brain and immunostained for GFAP and CD11b to confirm cellular identities. Square: enlarged image of the region. **b, c** Viability was measured by MTT assay (b) and cell counting (c). **d, e** Proliferation was evaluated using BrdU staining (d) and quantification of BrdU-positive cells (e). Viability was measured by MTT assay (f) and cell counting (g) after OGD/R. **f, g** Astrocytes were oxygen-glucose deprived for 6 h. After reoxygenation and treatment with vehicle or CU06-1004 (10 μg/mL) for 16 h, cells were added MTT for 48 h (f) or counted (g). **h, i** Proliferation was evaluated using BrdU staining (h) and quantification of BrdU-positive cells after OGD/R (i). Scale bars: 50 μm. $^{**}P < 0.01$. Error bars indicate mean ± SEM. ns, not significant; DAPI, 4′,6-diamidino-2-phenylindole; GFAP, glial fibrillary acidic protein; BrdU, bromodeoxyuridine; Con, control; OGD/R, oxygen glucose deprivation/reperfusion; 1004, CU06-1004

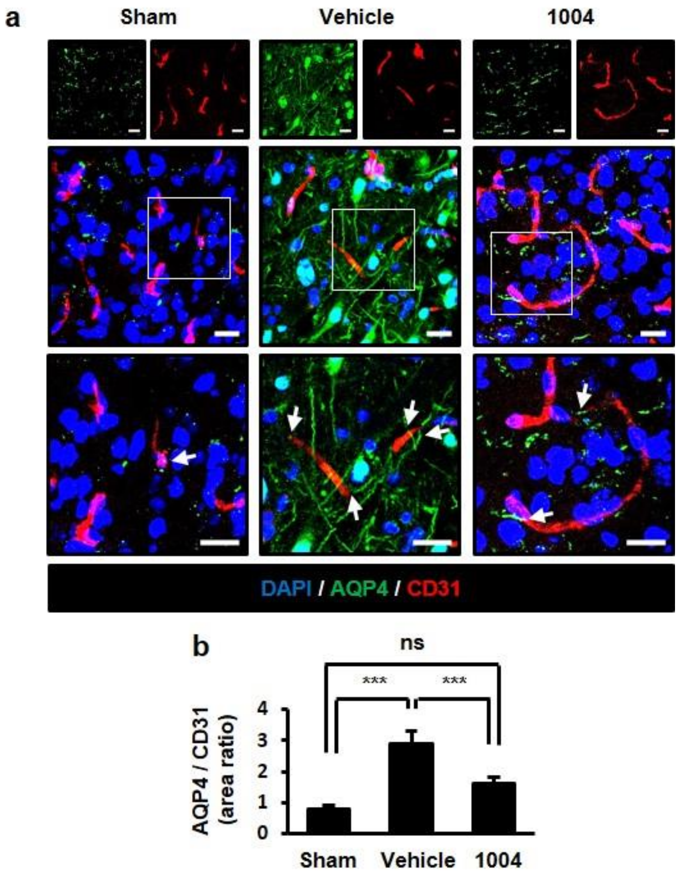

Fig. S4 CU06-1004 reduces increased AQP4 expression on blood vessels after I/R.

a Comparison of AQP4 expression in the sham, vehicle-I/R, and CU06-1004-I/R groups. Square: enlarged image of the region. Arrows show the presence of AQP4 on blood vessels. **b** Quantification of AQP4-positive blood vessels. n = 5 per group; scale bars: 20 μm. ***P < 0.001. Error bars indicate mean ± SEM. ns, not significant; DAPI, 4′,6-diamidino-2-phenylindole; AQP4, aquaporin 4; CD31, cluster of differentiation 31; 1004: CU06-1004

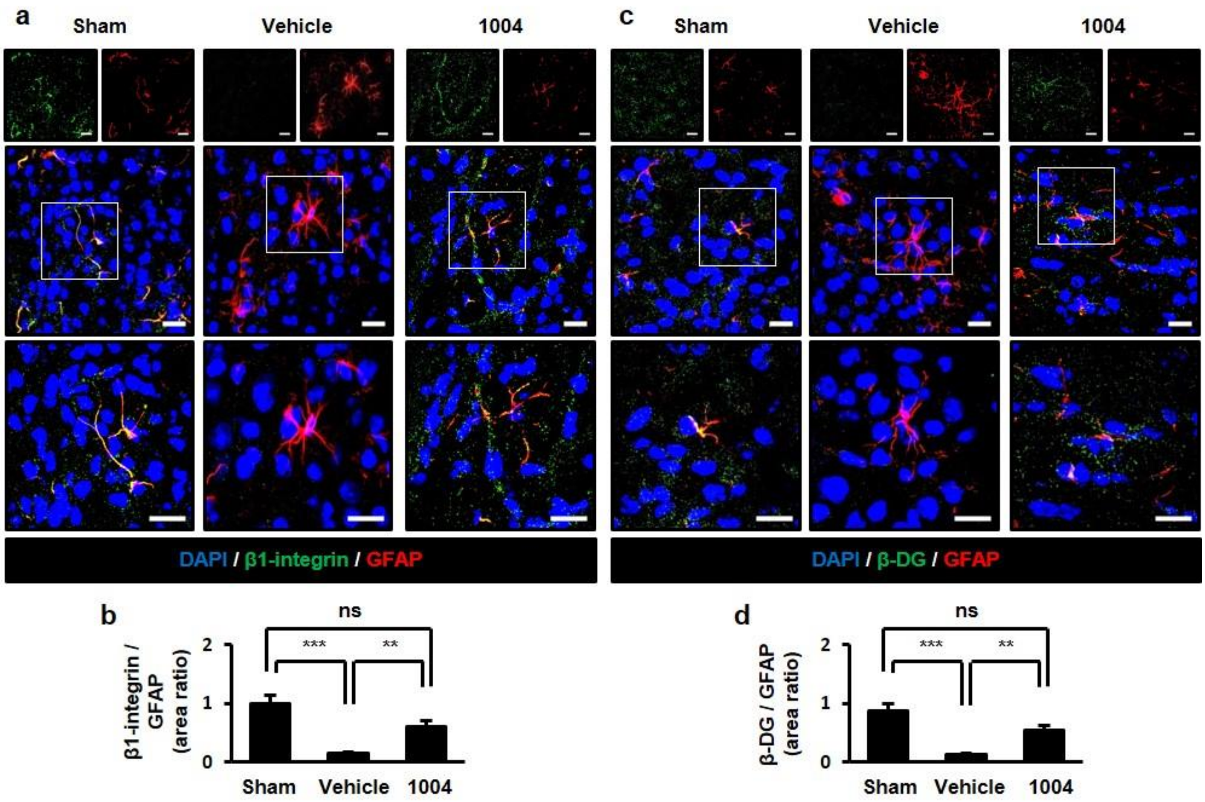

Fig. S5 CU06-1004 prevents the degradation of basal membrane proteins in astrocytes after I/R. **a, c** Comparison of β1-integrin (a) and β-DG (c) expression in the sham, vehicle-I/R, and CU06-1004-I/R groups. Square: enlarged image of the region. **b, d** Quantification of β1-integrin- (b) and β-DG-positive (d) astrocytes. n = 5 per group; scale bars: 20 μm. **P < 0.01 and ***P < 0.001. Error bars indicate mean ± SEM. ns, not significant; DAPI, 4′,6-diamidino-2-phenylindole; GFAP, glial fibrillary acidic protein; DG, dystroglycan; 1004, CU06-1004

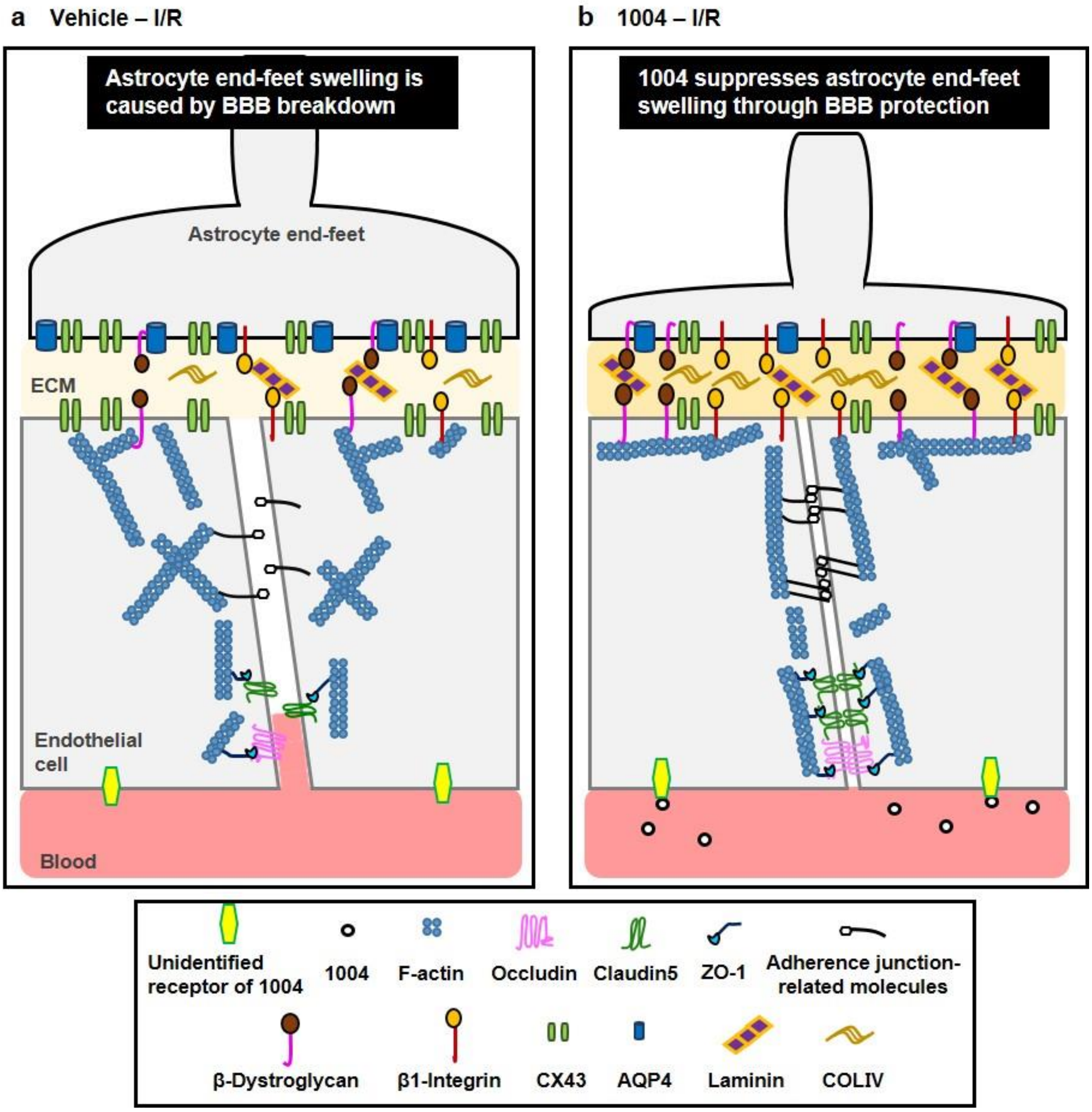

Fig. S6 Schematic of CU06-1004 function in astrocyte end-feet swelling after I/R.

a Following I/R, astrocyte end-feet are swollen by destabilization of cortical actin ring in ECs and BBB breakdown. Basal membrane proteins and ECM were disrupted by BBB breakdown. **b** CU06-1004 inhibits astrocyte end-feet swelling by suppressing the destabilization of cortical actin ring in ECs. Basal membrane proteins and ECM were also inhibited by CU06-1004. I/R, ischemia/reperfusion; F-actin, fibrous actin; CX43, connexin 43; AQP4, aquaporin 4; COLIV, collagen type IV; ZO-1, zona occludense-1; ECM, extracellular matrix; DG, dystroglycan; 1004, CU06-1004
