# Supplementary material for: CU06-1004 (endothelial dysfunction blocker) ameliorates astrocyte end-feet swelling by stabilizing endothelial cell junctions in cerebral ischemia/reperfusion injury
Source: J Mol Med (Berl). 2020 May 16;98(6):875–86. doi: 10.1007/s00109-020-01920-z (PMC7297708; doi:10.1007/s00109-020-01920-z)
Supplement: Supplementary file 2 — (PDF 274 kb) [file 109_2020_1920_MOESM2_ESM.pdf]

The endothelial dysfunction blocker CU06-1004 ameliorates astrocyte end-feet swelling by stabilizing endothelial cell junctions in cerebral ischemia/reperfusion injury

Dong Young Kim[1], Haiying Zhang[1, 2], Songyi Park[1], Yeaji Kim[1], Cho-Rong Bae[1], Young-Myeong Kim[3], Young-Guen Kwon[1]

[1]Department of Biochemistry, College of Life science and Biotechnology, Yonsei University, Seoul, Republic of Korea

[2]CURACLE Co., Ltd., Seongnam-si, Gyeonggi-do, Republic of Korea

[3]Department of Molecular and Cellular Biochemistry, School of Medicine, Kangwon National University, Chuncheon-si, Gangwon-do, Republic of Korea

Corresponding author

Young-Guen Kwon, Ph.D.

Department of Biochemistry, College of Life Science and Biotechnology, Yonsei University,

50 Yonsei-ro, Seodaemun-gu, Seoul, 03722, Republic of Korea

Tel: 82-2-2123-2903; Fax: 82-2-362-9897

E-mail: ygkwon@yonsei.ac.kr

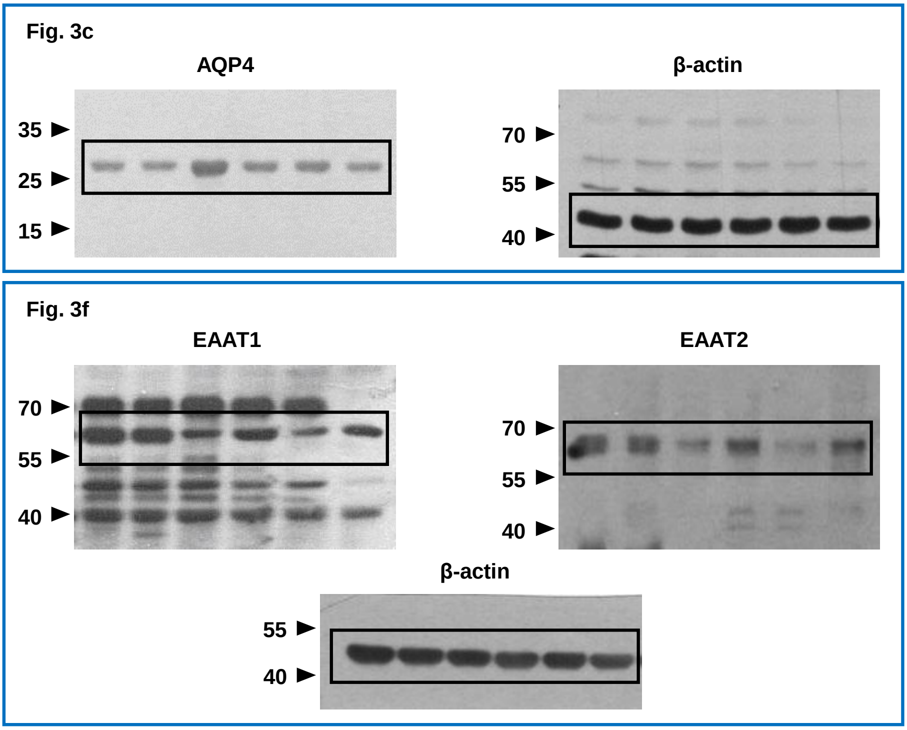

Full unedited gels for Figure 3

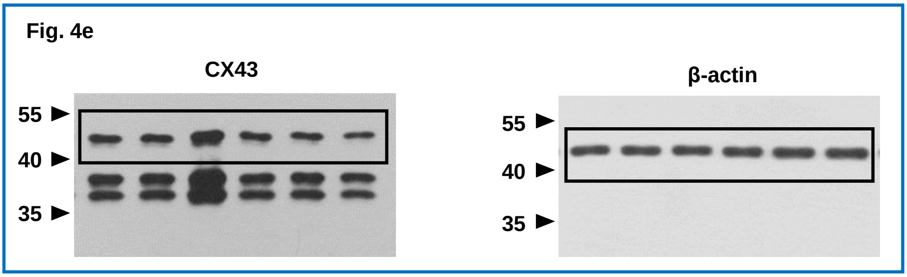

Full unedited gels for Figure 4

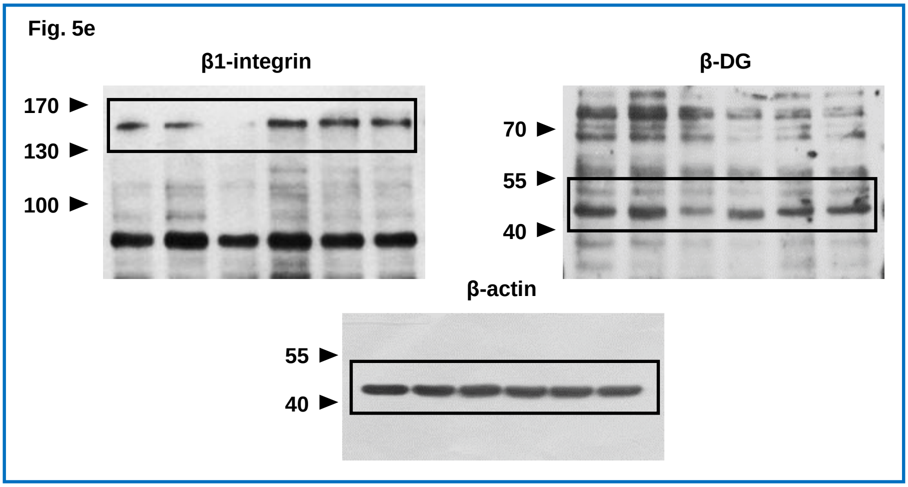

Full unedited gels for Figure 5

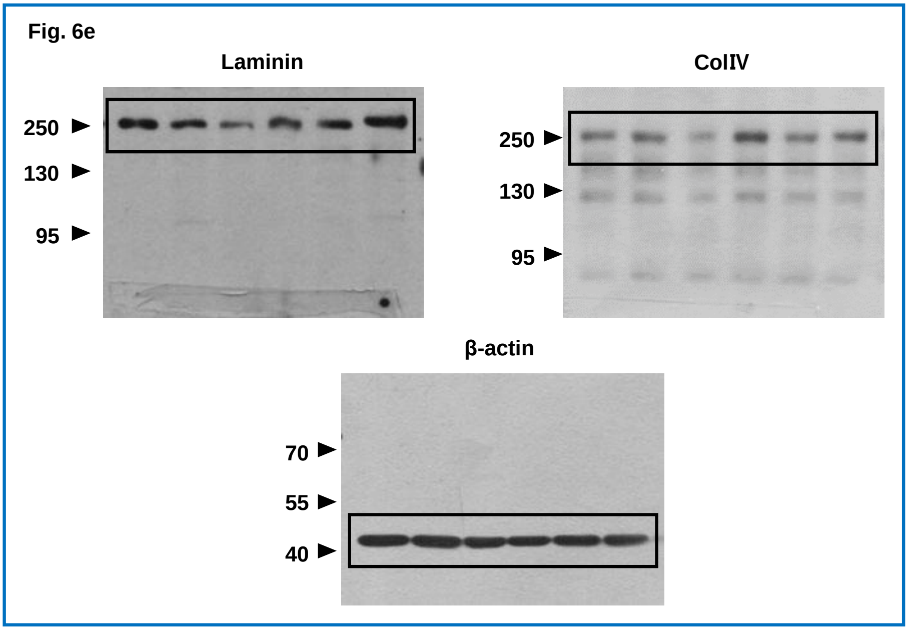

Full unedited gels for Figure 6

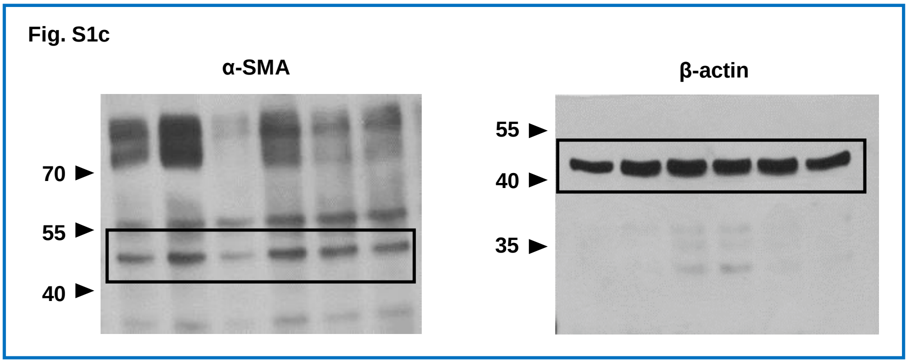

Full unedited gels for Supplemental Figure 1
